# Physicochemical Properties and Volatile Profile of Chito: A Traditional Dry-Cured Goat Meat Product

**DOI:** 10.3390/foods14132341

**Published:** 2025-07-01

**Authors:** Luz Hermila Villalobos-Delgado, Yaneisy Y. Martínez-Martínez, Guadalupe Virginia Nevárez-Moorillón, Joaquín T. Santiago-Castro, Sergio Soto-Simental, Carlos Ignacio Juárez-Palomo, Paula Cecilia Guadarrama-Mendoza

**Affiliations:** 1 Instituto de Agroindustrias, Universidad Tecnológica de la Mixteca, Av. Dr. Modesto Seara Vázquez No. 1. Acatlima, Heroica Ciudad de Huajuapan de León 69004, Oaxaca, Mexico; yedaniyaneisymar@gmail.com (Y.Y.M.-M.); tenochsc@mixteco.utm.mx (J.T.S.-C.); pcgm@mixteco.utm.mx (P.C.G.-M.); 2 Facultad de Ciencias Químicas, Universidad Autónoma de Chihuahua, Circuito Universitario s/n, Campus II, Chihuahua 31125, Chihuahua, Mexico; vnevare@uach.mx; 3 Instituto de Ciencias Agropecuarias, Universidad Autónoma del Estado de Hidalgo, Ave. Universidad s/n Km 1, Ex Hacienda de Aquetzalpa, Tulancingo 43600, Hidalgo, Mexico; sotos@uaeh.edu.mx; 4 Centro de Investigación y de Estudios Avanzados del IPN, Km. 9.6 Libramiento Norte Carretera Irapuato León, Irapuato 368241, Guanajuato, Mexico; carlos.i.juarez@cinvestav.mx

**Keywords:** chito, goat, ruminant meat, dry-cured meat products, TBARS, volatile compounds

## Abstract

Two types of chito were evaluated: non-pressed (NP, immediate consumption) and pressed (P, for sale). The characteristics were analysed in samples of three years (2021–2023). The pH, water activity (a_w_), proximate composition, heme iron, sodium chloride (NaCl), water soluble nitrogen (WSN), color, metmyoglobin (MMb), texture, lipid oxidation (Thiobarbituric acid reactive substances, TBARS), and microbiological analysis were evaluated, while volatile compounds were identified in NP and P. The a_w_ value showed a mean value of 0.70 in NP and P, values reported for typical commercial dried meat samples. However, P showed higher pH values (5.65–5.75), as well as a high level of fat (6.44–15.03%), NaCl (10.93–11.21%), lipid oxidation (3.88–6.32 mg MDA/kg meat), and hardness (223.67–574.01 N), with a browner color than NP, whereas microbial counts were similar between NP and P. Typical breakdown products derived from lipid oxidation were the main volatile compounds detected in chito, with aldehydes and alcohols being the most detected in P. The results suggest that some of the physicochemical characteristics, as well as the volatile profile, showed some differences between both types of chito, which suggests that there was a variation in the meat product associated with the making processes.

## 1. Introduction

Dry-curing is the oldest method employed for traditionally preserving meat products against the threat of spoilage and pathogenic organisms [1]. Nowadays, various dry-cured meat products are based on raw materials and processing conditions [2]. For example, “pastirma” in Turkey, “carne de sol” in Brazil, “kaddid” and “biltong” in north and south Africa, respectively, “jerky” in North America, “bresaola” and “violin de capra” in Italy, as well as “cecina” in Spain and Mexico, are just some of the many types of dried meat products [3,4,5].

Goat meat is usually transformed by drying, salting, and smoking to extend its shelf life. These processes aim to reduce enzymatic activity and delay the oxidation of fats and microbial attacks. This meat is accepted around the world; however, cultural and social traditions, along with economic conditions, frequently impact consumer choices [6].

Chito is a traditional salted and sun-dried goat meat product processed mainly in the states of Puebla and Oaxaca, Mexico. In general, the production process involves the use of meat from the goat’s forearm, hips, breast, and neck. Firstly, this meat is salted in vats containing sea salt for 4 h at room temperature and covered with palm mats. After salting, the meat strips are placed on palm mats and dried in the sun for 3 or 4 days until the desired texture is achieved by the “chitero” (worker who processes chito); the texture is hard, similar to frozen meat. Two types of chito can be obtained: non-pressed for immediate consumption and pressed, which is destined for sale in other states. Chito that is destined for sale in other states is pressed with mallets made from sabino or oak wood and packed in jute bags.

Considering the trend of increased production and consumption of goat meat worldwide, the objective of this research was to evaluate for the first time the physicochemical and microbiological characteristics as well as identify the volatile compounds of chito (non-pressed and pressed) over three consecutive years (2021, 2022, and 2023). The goal was to provide a scientific foundation for enhancing the quality of this intermediate moisture meat product.

## 2. Materials and Methods

### 2.1. Sample Collection

The processes for preparing the meat product were not established, and only the prepared product was analysed. Chito samples were acquired within 2 days after processing during the autumn season (October and November). For laboratory analyses, two types of chito, non-pressed (NP; immediate consumption) and pressed (P; for sale), were randomly purchased (packed in sealed plastic bags) from hacienda “El Rosario”, located in Huajuapan de León, Oaxaca, Mexico. The chito samples (NP and P) (400 g) consisted of meat strips from approximately 10 to 15 cm long and approximately 4 cm thick obtained from goat forearm, hips, breast, and neck. To determine the quality of the chito, the samples were evaluated annually over a three-year period (2021, 2022, and 2023). The samples were taken to the laboratory and divided into two portions. The first portion was used for pH, water activity, proximate composition, heme iron content, sodium chloride, water soluble nitrogen, color measurement, metmyoglobin content, texture measurement, lipid oxidation, and microbiological analysis. The other portion was vacuum packaged and frozen (−40 °C) until further analysis (volatile compounds).

### 2.2. Analysis of the Meat Product

#### 2.2.1. Physicochemical Analysis

Before analysis, the samples were ground in a multiprocessor (Hamilton Beach Brands, Mexico City, Mexico) until complete homogenization of the meat was achieved. The pH for each sample was measured by homogenizing a 10 g sample with 90 mL Milli-Q water using an Ultraturrax T18 (IKA, Staufen, Germany) for 60 s. The measurements, in triplicate, were performed with a pH meter (HANNA instrument, Woonsocket, RI, USA) calibrated using standard buffers with pH 4 and 7 (J.T. Baker, Xalostoc, Mexico) [1]. Water activity (a_w_) was measured using HigroPalm model HP23-AW (Rotrotonic^®^ Instrument Corp, NY, USA). The sodium chloride content was determined according to the Carpentier–Vohlard official method [7]. Moisture, protein, fat, and ash contents were determined following the Official Methods number 950.46, 981.10, 991.36, and 920.153, respectively [8]. Water-soluble nitrogen (WSN) was determined on a supernatant obtained following the procedure by Villalobos-Delgado et al. [9]. The lipid oxidation degree of the samples was determined using the TBARS (thiobarbituric acid reactive substances) method of Villalobos-Delgado et al. [10]. A 2 g meat sample was homogenized in 20 mL of Milli-Q water with an ultraturrax T18 (IKA, Staufen, Germany) at 9000 rpm for 1 min. Then, 1 mL of meat homogenate was mixed with 30 mL of butylated hydroxytoluene (Sigma Aldrich, MO, USA) [7.2%, in ethanol (J.T. Baker, Xalostoc, Mexico)] and 2 mL of thiobarbituric acid:trichloroacetic acid solution [20 mM TBA (Alfa Aesar, MA, USA) and 15% (*w*/*v*) TCA (Sigma Aldrich, MO, USA)]. The mixture was vortexed (IKA, Staufen, Germany) and then incubated in a water bath (Riossa, Mexico City, Mexico) at 80 °C for 20 min. After cooling for 10 min in cold water, the sample was centrifuged (Eppendorf 5804 R, Hamburg, Germany) at 2500 rpm for 10 min at 4 °C. The absorbance was measured at 531 nm, and the TBARS values were determined from a malondialdehyde (MDA) standard curve with 1,1,3,3-tetraetoxypropane (TEP) (Sigma Aldrich, MO, USA) and were expressed as mg MDA/kg sample.

#### 2.2.2. Color Measurement

Meat color was determined on the surface of chito using the lightness (L*), redness (a*), and yellowness (b*) system with a UltraScan Vis 1139 colorimeter configured to operate with an aperture of 9.525 mm, a 10° observer angle, and a D65 illuminant (HunterLab, VA, USA). The hue (H*) and chroma (C*) parameters were determined using the following equations: H* = [tan − 1(b*/a*) × (180/π)]; and C* = [(a*^2^ + b*^2^)^½^] [11].

#### 2.2.3. Metmyoglobin (MMb) and Heme Iron Content

The metmyoglobin content of the chito (NP and P) was determined as described by Shi et al. [12], with some modifications. The minced chito samples (5 g) were mixed with 25 mL ice-cold sodium phosphate buffer solution (pH 6.8; 40 mM) (Meyer, Mexico City, Mexico). The mixture was homogenized for 60 s at 9000 rpm/min. The homogenized sample was maintained at 4 °C for 1 h and then centrifuged at 6400 rpm for 30 min at 4 °C. For each sample, the supernatant was filtered through a Whatman No.1 filter, and the absorbance was read at 525, 545, 565, and 572 nm by a spectrophotometer. The percentage for the metmyglobyn was determined using Formula (1) as follows:

(1) $$\mathrm{MMb}\;\left(\%\right)=\left[-2.514\;\left(\frac{A_{572}}{A_{525}}\right)+0.772\left(\frac{A_{565}}{A_{525}}\right)+0.8\left(\frac{A_{545}}{A_{525}}\right)+1.098\right]\times 100$$

The heme iron content of the chito was determined according to the methodology described by Cheng and Ockerman [13], with some modifications. From the minced meat, 2.5 g was mixed with 10 mL of acetone (Meyer, Mexico City, Mexico), 0.5 mL Milli-Q water, and 0.25 mL concentrated hydrochloric acid (J.T. Baker, Xalostoc, Mexico). The sample was mixed with a glass stirring rod and was then sealed and kept in the dark for 1 h. The sample was filtered through Whatman No.1 filter paper and the filtrate was measured at 640 nm. The heme iron content of the chito was obtained via the following Formula (2):

(2) $$\mathrm{Heme}\;\mathrm{iron}\;\mathrm{content}\;\left(µg/g\right)=A_{640}\;\times \;680\;\times \;0.0882$$

where *A*_640_ is the absorbance of the filtrate at 640 nm.

#### 2.2.4. Instrumental Texture Measurement

Instrumental Texture Profile Analysis (TPA) [14] was carried out using a texture analyzer TA TXPlus (Stable Microsystems, Surrey, UK) with a cylindrical probe P25P set to run at 1 mm/s. Six cubes of chito (1 cm each side) were compressed twice (50% of their original height) with a 1 s interval between the two compression cycles. The tests were undertaken at room temperature, and the parameters determined from the force–time curves (software Exponent ver. 6.1.20.0) were hardness, springiness, cohesiveness, and resilience.

#### 2.2.5. Microbiological Analysis

Meat is normally assumed to be contaminated with a range of bacteria and moulds, which include both spoilage and pathogenic. Total mesophilic bacteria count (TMBC), total coliform count, the total count of yeast and moulds, lactic acid bacteria, *Salmonella* spp. (Gram-negative pathogen), and coagulase-positive staphylococci (Gram-positive pathogen) were determined following standard methods [15].

For microbial counts, 25 g of each sample was obtained aseptically and homogenized for 2 min with 225 mL of peptone water using a homogenizer (BagMixer Interscience Model CC, Saint Nom, France). Serial 10-fold dilutions were then prepared and used to inoculate agar plates by the pour plate method for the enumeration of microbial groups. The total mesophilic bacteria count (TMBC) was enumerated in plate count agar (PCA) media (BD, Bioxon, Mexico City, Mexico) following incubation at 30 °C for 48 h. Lactic acid bacteria (LAB) were inoculated in de Man-Rogosa-Sharpe (MRS) agar (BD Bioxon, Mexico City, Mexico) and incubated at 37 °C for 48 h under a reduced oxygen atmosphere (BD Gas Pack CO2, Franklin Lines, NJ, USA). Yeast and moulds were enumerated on acidified potato dextrose agar (PDA) (Bioxon, Mexico City, Mexico) (supplemented with 1.5 mL/100 mL of 10% *w/v* tartaric acid), which was incubated at 25 °C for 5 days. To determine the enumeration of total coliforms, dilutions were inoculated in Violet Red Bile Agar (VRBA) (Bioxon, Mexico City, Mexico) and incubated at 37 °C for 24 h. For the determination of coagulase-positive staphylococci, 0.1 mL of each dilution was inoculated by the spread plate method on Baird Parker agar (BD Bioxon, Mexico City, Mexico) and incubated at 37 °C for 48 h [15]. Microbiological data were transformed into logarithms of the number of colony-forming units (CFU/g).

The procedure for determining the presence of *Salmonella* spp. in the meat samples was followed according to Mexican legislation [16]. A 25 g sample was homogenized in 225 mL of buffered peptone water, and the mixture was incubated at 37 °C for 18 h. After incubation, 1 mL was transferred to tubes with 9 mL of RVS broth (Rappaport-Vassiliadis Soy Borth) (Bioxon, Mexico City, Mexico) and MKTTn (Muller-Kauffmann Tetrathionate added with novobiocin) (Bioxon, Mexico City, Mexico) and incubated at 37 °C for 24 h. After incubation, a loopful was inoculated into XLD (Xylose Lysine Deoxycholate) agar and Bismuth Sulfite Agar (Bioxon, Mexico City, Mexico) and incubated at 37 °C for 24 h.

#### 2.2.6. Analysis of Volatile Compounds

Volatiles were extracted from chito samples using headspace solid phase microextraction (HS-SPME GC-MS) with a DVB/CAR/PDMS-50/30 µm fibre according to Villalobos-Delgado et al. [9] and Pavlidis et al. [17], with some modifications. In a 20 mL headspace vial (Agilent Technologies, CA, USA), 2.5 g of chito sample (previously ground) was mixed with 5 mL of 25% NaCl solution (KEM, Naucalpan, Mexico) for 1 min. Then, 3 µL of 3-octanol solution (Fluka Chemika, Buchs, Switzerland) (0.3 µL/mL methanol) (Tedia Company, Fairfield, OH, USA) was added as an internal standard, and the vial was sealed using screw caps with silicone/PTFE septa (Agilent Technologies, USA) along with a mini-nert valve.

Prior to the injection, the vial containing the sample was equilibrated at 90 °C for 30 min in agitation. Then, the SPME fibre was exposed to the sample for another 30 min under the same conditions with continuous agitation to maximize the adsorption of analytes. Immediately, the fibre was retracted and inserted into the inlet injection port of a GC 7890B gas chromatograph (Agilent Technologies, CA, USA) for desorption for 7 min at 260 °C.

Afterwards, volatiles were analysed using an HP 7890B gas chromatograph (Agilent Technologies, CA, USA) equipped with an HP-5MS column (30 m × 0.25 mm × 0.25 µm; Agilent Technologies, USA) directly coupled to an HP 5977a mass-selective detector. Helium served as the carrier gas at a constant flow rate of 1 mL/min in splitless mode. The needle temperature was 250 °C. The column’s initial temperature was set at 40 °C for 1 min with a ramp of 15 °C/min until it reached 250 °C; the temperature was maintained for 1 min. The mass spectrometer was operated in electron impact mode (70 eV) with a scanning range of 30–550 *m*/*z*. Volatiles were identified by matching their retention times and mass spectra with those from authentic compounds available from computerized spectral databases (NIST/EPA/NIH 75 K), analyzing the spectrum, and ensuring a match equal to or greater than 80%. All concentrations of identified volatile compounds were reported as µg/kg.

#### 2.2.7. Odor Activity Value (OAV) Analysis

To evaluate the contribution of each volatile compound to the odor profile of chito (NP and P), the OAV was calculated as the ratio of the concentration of volatile compounds to their threshold value. Compounds with OAV > 1 influenced the odor profile [18].

### 2.3. Statistical Analysis

All analyses were conducted in triplicate across three batches of samples, and the results were presented as means with their corresponding standard errors of the mean (SEM). For all variables considered in this study, statistical analysis was undertaken with one-way analysis of variance (ANOVA) using the Statistica software (version 10). The mean values for each parameter (individual trait) were compared across different sampling times (years 2021, 2022, and 2023) in each type of meat and between samples (NP and P) in each sampling time. Differences between means in the obtained results were tested for significance (*P* < 0.05) using Duncan’s multiple range test. Using the concentrations of the compounds with an OAV > 1, a Partial Least Squares Discriminant Analysis was performed using the MetaboAnalyst 6.0 platform (https://www.metaboanalyst.ca, accessed on 23 June 2025 to obtain the VIP score graphic.

## 3. Results and Discussion

### 3.1. Physicochemical Characteristics

The physical and chemical characteristics (pH, a_w_, proximate composition, heme iron, NaCl, and water-soluble nitrogen) of chito are shown in Table 1.

For each different year, pH, moisture, fat, heme iron, and NaCl were significantly affected by pressing (*P* < 0.05), except for moisture, fat, and heme iron in 2021. For each year, the pH values of P showed a small but significant increase (*P* < 0.05) compared to NP. The pH values ranged from 5.08 to 5.28 in NP and from 5.65 to 5.75 for P. Due to the short processing period, the increase in P could be partially attributed to the effect of drying and pressing. Mediani et al. [4] reported that the pH of dried meat products is affected by drying procedures due to protein denaturation. In this sense, the increase in pH values in P could be attributed to the reduction of acidic groups present in meat due to conformational changes associated with protein denaturation (through moisture loss) [19] and the force applied to the meat by pressing. Thus, the average values of pH for NP and P are partially in agreement with Mediani et al. [4], who reported that the pH of commercial dried meat samples ranged from 5.4 to 5.8. Moreover, in this study, pH values below 5.4 were observed; a low pH is important for avoiding the denaturation of protein in the meat.

On the other hand, the mean a_w_ values of NP and P (a_w_ < 0.70) fall within the range reported for so-called intermediate moisture meat products (0.60–0.90) [9]. Values under 0.75 are relatively safe against microorganisms during storage at ambient temperature [20]. In general, the salting processes not only help with drying but also reduce the a_w_ values of meat. Salt penetration into the meat along with water loss from the tissue are simultaneous processes.

Furthermore, for the years 2022 and 2023, the pressing significantly (*P* < 0.05) decreased the moisture content and heme iron, whereas the fat and sodium chloride (salt) content increased. The fall in moisture content is initially attributed to the pressing, which could have released tissue fluids to the exterior of the meat, promoting drying and water loss. Additionally, chito thickness could have contributed to the diffusion of salt and facilitated drying. Salt is used for dehydrating meat, which involves removing water from the meat in a non-thermal manner [4], which implies a reduction in the water activity and myoglobin concentrations [21], leading to a decrease in heme iron content. The sodium chloride content found in the chito was comparable to that reported in beef cecina (10% NaCl), which is an intermediate meat product produced and consumed mainly in Mexico [5]. Regarding the reduction in heme iron content in pressed chito, it may have implications for its nutritional value, as iron from animal foods is more bioavailable than iron from plant sources. In similar studies, Shi et al. [12] noticed that, during hot air drying of beef jerky, the heme iron content decreased. This effect has been attributed to oxymyoglobin oxidation and a reduction in metmyoglobin. Furthermore, Zdanowska-Sąsiadek et al. [22] observed that adding salt and then spices reduced heme iron content in dried ostrich meat.

Furthermore, statistical analysis showed that, in 2022, NP and P exhibited higher (*P* < 0.05) fat content and heme iron content than in the years 2021 and 2023. In the case of fat, particularly in P treatment, the pressing and a higher loss of moisture could have had an impact on the highest fat content; a decrease in the humidity allows the concentration of fat. However, for the year 2021, the fat content in the chito (NP and P) is comparable to that reported by Paleari et al. [6] and Teixeira et al. [21], who found values of 7.14% and 6.03% from salted-ripened goat thigh and cured goat legs, respectively. Finally, for WSN, NP and P showed higher values in 2021 than in 2022 and 2023. The result for NP corresponds with those reported by other authors [9] in similar dry-cured ruminant products.

### 3.2. Instrumental Color and Metmyoglobin Content

The results for instrumental color and metmyoglobin content of chito are shown in Table 2. Statistical analyses for each different year did not reveal any significant differences (*P* > 0.05) in L*, b*, and chroma between both treatments. However, for 2021 and 2023, NP showed higher values than P (*P* < 0.05) for a*. Similarly, a* values (7.47) were noticed by Ortega et al. [23] from salted goat after the air-drying process. In contrast, for these same years, P showed values higher than NP for Hue. These results suggest that P had lower redness (larger angles are more yellow) because of the oxidation of myoglobin as well as the formation of metmyoglobin (MMb) [11] due to salting and drying. Guo et al. [24] argued that, in an aerobic environment, myoglobin is oxidized to MMb, which suggests that iron in heme is oxidized to a ferric state in dried meat, making the meat darker [4,12]. Additionally, the Maillard reaction impacts the color of dried meat products and occurs between the reducing sugars’ carbonyl groups and free amino acids in the muscle over the course of the drying period. Ribose is a reducing sugar and is closely related to the degree of browning in dried meat products [25]. In spite of these results, the metmyoglobin content for NP and P was not different (*P* > 0.05) for the years 2021 and 2023. However, for 2022, P showed the highest MMb content. This finding coincides with data reported by Shi et al. [12], who found values within the range of from 50 to 55% of MMb when beef jerky was evaluated during hot air gradient drying.

In contrast, significant differences (*P* < 0.05) were detected for the L*, a*, Hue, and metmyoglobin content values between different years. In this regard, L* values ranged from 20.31 to 25.92 in NP, whereas, in P, they ranged between 20.11 and 26.79. These findings fall within the range reported (20–50) for L* in dried meat [4]. In addition, similar L* values (25.27) were noticed by Ortega et al. [23] from salted goat. Regarding a* values, the year 2022 showed the lowest values (less red) in both treatments, whereas NP showed the highest values in the Hue angle. Moreover, 2022 showed higher values in MMb content compared to 2021 and 2023.

### 3.3. Texture Evaluation

Table 3 shows the results on the instrumental texture of chito. For each year, no significant differences (*P* > 0.05) were observed in springiness, cohesiveness, and resilience between the treatments. On the other hand, mean hardness was higher in the P treatment than in NP for each year. Moisture loss, protein denaturation, and the shortening of fibres during meat drying, as well as collagen and fat content, result in the formation of an intense and hard structure [2,4,25]. In this regard, the results of this study could be attributed to various factors. Firstly, some of the meat samples were extremely non-homogeneous regarding shape and localization of fat. However, the main factor could be attributed to the effect of pressing with mallets (high force) on dried meat (before packing), which compacted and hardened the meat even more, thereby changing the consistency from firm to hard. Moreover, it is worth mentioning that this may also be related to muscle types, anatomical location, and the age of the animal. In addition, the drying process can reduce the size of the meat pieces and, to some degree, wrinkle them. Thus, dried meat products develop a hardened texture and wrinkled appearance due to volume reduction, while the meat sometimes acquires a hard crust on the surface [26]. Aktaş et al. [27] argued that higher salt concentrations in the muscle may denature proteins, leading to stronger protein-protein bonds, muscle shrinkage, and dehydration.

Conversely, Reyes-Cano et al. [5] found that maturation significantly affects meat texture through proteolysis and other enzymatic processes. However, chito has a short processing time and, therefore, it does not allow for an intense action of muscle proteases on myofibrillar protein. Furthermore, their salt content could decrease their proteolytic activity due to chloride sodium concentrations above 8%, which could have completely inhibited muscle protease activity [20]. It should be noted that the hardness of the pressed samples is not a problem for the consumer, since chito is typically consumed in broth, where heat and hydration soften its texture [26].

Finally, between the different years, NP showed the highest values (*P* < 0.05) in hardness for 2022, whereas P showed a similar behavior for 2022 and 2023. For resilience, 2021 was the year that showed the highest resilience in NP and P compared with the other years, indicating that both samples had the ability to recover from deformation.

### 3.4. Lipid Oxidation

**Figure 1:**
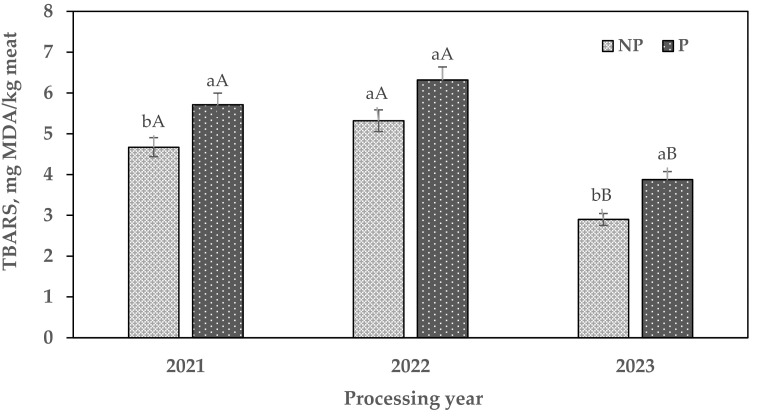
Mean values of lipid oxidation (TBARS, mg of malondialdehyde/kg of meat) in chito. NP: non-pressed (immediate consumption); P: pressed (for sale). ^a,b^: Means within the same year with different letters are significantly different (*P* < 0.05). ^A,B^: Means within the same treatment with different letters are significantly different (*P* < 0.05).

Figure 1 shows the lipid oxidation (TBARS) degree of chito. In 2021 and 2023, P treatment had higher TBARS values (*P* < 0.05) than NP, whereas, in 2022, there were no differences (*P* > 0.05) among the treatments. These results could be attributed to sodium chloride and fat content, as well as drying and pressing. Bermúdez et al. [28] reported that the increase in malondialdehyde (MDA) could be associated with the prooxidant effect of metallic ions present as impurities in the salt used in meat products such as dry-cured Celta ham.

Moreover, drying is performed in the open air, which accelerates the lipid oxidation process. Kim et al. [29] argued that, during the drying process, the evaporation of moisture results in fat exudating to the surface, which can enhance the contact between fat and oxygen, thereby promoting lipid oxidation. Thus, the TBARS values rise with an increase in fat content in the food [4]. On the other hand, comparing between years (considering the same treatment), in 2021 and 2022, NP and P treatments had higher (*P* < 0.05) lipid oxidation levels compared with 2023. For 2022, it is notable that the P treatment showed TBARS values of 6.32 mg MDA/Kg meat. This finding indicates that lipid oxidation occurred at the highest level in P for this year (2022), corresponding well with the highest fat and sodium chloride content found in this treatment, as well as the packaging, which consisted of jute sacks, which expose meat to atmospheric oxygen (the primary oxidizing agent). Thus, this finding differs from data previously reported by Teixeira et al. [21] and Oliveira et al. [30], who observed that cured goat legs and salted-ripened goat meat (mantas) presented lower TBARS values (4.96 and 1.87 mg of MDA/Kg, respectively). These results are also in contrast with what has been described in the literature for similar dry-cured meat products, such as alpaca charqui, where the mean values for TBARS were between 2.5 and 4 mg of MDA/Kg [20].

In this sense, it is known that the acceptable limit of the oxidation degree is <2 mg MDA/Kg [31], a value that is associated with the threshold of consumer detection of rancid flavor. However, this limit could not be considered as real due to some authors having argued different lipid oxidation detection or acceptability limits depending on the type of meat product [11,32]. Thus, some authors, such as Sampels et al. [33], indicated that TBARS values remain higher in dehydrated meat products compared to fresh and smoked meat products.

### 3.5. Microbial Counts

Analyses revealed that no evidence was found of *Salmonella* spp. in the chito samples. Furthermore, counts of moulds and yeasts, coliforms, and coagulase-positive staphylococci were under detectable levels (<10 CFU/g) in both samples. These results suggest that the processing of chito with sodium chloride and drying conferred a degree of stability and protection against these bacterial groups. In particular, Vilar et al. [34] and Heo et al. [35] argued that the salt-tolerant bacteria belonging to the Micrococcaceae family are usually isolated in raw-cured meat products, such as ham or Spanish beef cecina. These microorganisms can persist until the final manufacturing stages due to their high tolerance for salt (most strains survive in the presence of 10% NaCl) as well as their resistance to low a_w_ and high osmotic pressure conditions. However, in this study, the overall occurrence of coagulase-positive staphylococci in chito (NP and P) was low.

On the other hand, for each year, no significant difference (*P* > 0.05) between NP and P was observed for TMBC and LAB (Table 4). Furthermore, comparing between years, microbial analyses revealed that, for the LAB population, there were no significant differences (*P* > 0.05). However, TMBC was predominantly higher in 2022 and 2023 than in 2021 for NP (6.68, 6.62, and 5.92 log CFU/g, respectively), whereas the highest TMBC values were detected in 2022 for P (7.0 log CFU/g). These findings could be related to various post-slaughter factors, such as handling, processing, storage conditions, and packaging. It is worth mentioning that the level of TMBC found in chito (especially for 2021) was slightly higher than that obtained in a study previously carried out on fresh goat meat (5.6–5.8 log CFU/g) (unpublished data), supporting the theory of re-contamination during chito processing. Reyes-Cano et al. [5] found higher counts in beef cecina (intermediate moisture meat) (7.4–9.6 log CFU/g) coming from different states of Mexico than those recommended in the Mexican Official Standard [36] for raw meat (6.7 log CFU/g). For cecina produced in Oaxaca state, the count for mesophilic aerobic microorganisms was 30 × 10^6^ (7.4 log CFU/g). In addition, Petit et al. [1] also reported total microbial counts that ranged from 6.2 to 9.7 log CFU/g in commercial biltong samples. Thus, although chito could represent a risk (in view of its ease of contamination), cooking the meat product can decrease the number of microorganisms. The chito product is not usually consumed without thermal processing, as is the case with biltong, which can be eaten raw without prior rehydration and/or cooking.

### 3.6. Volatile Compounds

GC-MS results for volatile compounds were processed as shown in Table 5 and via the heat map (Supplementary Materials). A total of 78 volatile compounds were found in the chito samples. Volatiles were within the following seven groups: aldehydes (19), ketones (7), alcohols (9), acids (14), terpenes (17), hydrocarbons (9), and phenols (3), which generally aligns with previous research on the volatile profile of dry-cured ruminant meat products [6,9,20,37].

It can be observed that the detected compounds were different for each year by considering both samples. For 2021, compounds such as aldehydes and acids were detected, and only a small number of alcohols and phenol compounds were found. For 2022 and 2023, aldehydes, ketones, alcohols, acids, terpenes, and hydrocarbons accounted for the highest proportion. Aldehydes, alcohols, and hydrocarbons have been related to lipid degradation [9]. Regarding these volatile compounds, despite presenting very similar proportions between treatments, it could be observed that the P samples showed slightly higher proportions than NP. These results could be related to malondialdehyde content, in which P showed greater lipid oxidation than NP.

In this sense, Ivanovic et al. [37] reported that aldehydes are generally major sources of volatile fractions obtained from ruminant meat, which mainly originate from the oxidative degradation of unsaturated fatty acids, such as oleic, linoleic, and arachidonic [44]. These could significantly contribute to the product’s overall taste because of the low levels of olfactory perception [37]. Heptanal, octanal, nonanal, decanal, and decenal are primarily derived from the oxidative degradation of oleic acid [6,45,46], whereas hexanal and 2-nonenal are the main volatile compounds formed by the oxidation of linoleic acid. Additionally, hexanal comes from arachidonic acid, which has a pleasant and grassy aroma at low concentrations but turns fatty at medium concentrations and extremely rancid and tallowy at high concentrations [44]. Thus, high concentrations of hexanal indicate flavor deterioration in meat products that often result in a rancid aroma. Therefore, this volatile compound has been considered the most suitable indicator of lipid oxidation in meat and meat products, as there is a more significant increase in its content compared to that of other aldehydes [45]. Furthermore, octanal provides green, meaty, fresh, fruity, and grassy notes, whereas nonanal contributes fruity and sweet aroma notes [47]. Thus, the aldehydes detected, such as hexanal and nonanal, likely have an impact on chito’s sensory profile, as these compounds are associated with grassy and fatty notes in cured meat products.

On the other hand, ketone detection in meat usually correlates with dietary habits [37]. However, Paleari et al. [6] reported that ketones, such as 2-heptanone and 2-nonanone, are most likely of chemical origin and are produced through either oxidation or thermal degradation of the fatty acids, or by degradation of amino acids. For 2022 and 2023, it was found that 2-heptanone and 2-nonanone were present in chito. Thus, its presence can be explained by dietary origin (goats were fed grass and different wild herbs) and the oxidation of fatty acids.

Furthermore, straight-chain aliphatic alcohols can be formed by the oxidation of lipids [44]. Among the alcohols, 1-octen-3-ol was present during 2021, 2022, and 2023 in both chito samples. This alcohol is the major alcohol in meat products and is generated by the catabolism of both arachidonic and linoleic acids [47]. It possesses a low perception threshold and is characterized by odors resembling those of mushrooms, earth, dust, fatty substances, sharpness, and rancidity [44]. Other compounds detected in the volatile composition of chito were acids, which are responsible for the distinct taste of goat meat [37], such as 3 methylbutanoic, which contributes to the goaty flavor [48], but it was not detected in this study. This group of compounds is formed by the hydrolysis of triglycerides and phospholipids and primarily from the oxidation of unsaturated fatty acids [44].

Additionally, terpenes may significantly contribute to this meat product’s overall aroma. Their presence may be the result of diet, which leads to terpene accumulation in the animal’s fat deposits [6]. Terpenes primarily come from green herbages and are normally too specific to certain plant species for use as generic tracers of a pasture diet [49]. In this sense, terpenes such as caryophyllene were detected in NP and P for 2021, 2022, and 2023. Caryophyllene content in meat is influenced by pasture grass diets; hence, it is a biomarker of non-intensive feeding. Moreover, other terpenes, such as D-limonene, were also detected in both chito samples. Limonene is present as a normal constituent of the unsaponifiable fraction of vegetal fats and originates from animal feed, thereby accumulating in the meat of the animal [6], i.e., in adipose tissues of pasture-fed lambs. This compound gives the smell of fresh fruit (conveyed by lemon) [6,49]. Furthermore, p-cymene, copaene, and β-caryophyllene are classified as recurrent pasture diet tracers in the literature and can be considered as generic pasture diet tracers [49]. Sohail et al. [38] reported that some phenolic compounds can arise from the digestion of plants. In this sense, ruminants whose ruminal microflora contain ligninase and associated digestive enzymes can produce phenol and *p*-cresol from lignin.

On the other hand, hydrocarbon compounds were detected in 2022 and 2023, whereas these same compounds were not detected in 2021. Hydrocarbons can be formed by lipid autooxidation processes, which may be catalysed by heme compounds, such as hemoglobin and myoglobin, or by the decomposition of carotenoids [6,44]. Among the hydrocarbons identified, heptane and octane are believed to originate primarily from the oxidation or degradation of polyunsaturated fatty acids [20]. Furthermore, pentadecane, hexadecane, and heptadecane have been detected as reliable pasture diet tracers [49]. Moreover, phytane (isoprenoid hydrocarbon) is a typical compound in meat from ruminants allowed to graze [20]. In this sense, the feeding of the goats from which the chito comes was carried out under an extensive grazing system involving grass and different plants in the Mixteca Oaxaqueña region. However, at the sensory level, hydrocarbons may not be important contributors to meat flavor as they have relatively high odour threshold values [3,6,44].

Finally, benzene compounds, such as benzaldehyde, and other compounds, such as phenols, were detected only in 2021 in both chito samples. The presence of benzaldehyde suggests it is linked to lipid oxidation [9]. The other compounds include phenol, *p*-cresol, and m-cresol, which have been detected in smoked cured meats and are considered the main constituents of the typical smoky aroma and flavor [50]. They occur either in a gaseous state or condensed into particles, or both. In this study, their presence could be considered a constituent of caprine fats (*m*-cresol and *p*-cresol) [51] or as a contaminant (chito was not smoked). In this regard, it is possible that, during the processing of chicharron of goat (meat co-products are fried to a golden-brown color), these compounds could have been generated and then migrated from the environment to the surface of chito as particles of smoke.

### 3.7. Odor Activity Value (OAV)

The OAV represents the contribution of a compound to the overall odor. Thus, a higher OAV indicates a stronger contribution of the compound to the aroma characteristics [52]. Table 6 shows variations of volatile compounds with OAVs > 1. Twenty-four key odor compounds with OAVs of >1 were detected in NP and P, of which the most important were aldehydes (11), ketones (5), alcohols (5), and terpenes (3). Zhang et al. [18] and Dominguez et al. [45] reported that alcohols and aldehydes are highlighted as the primary volatile compounds responsible for the dominant odor in meat. Notably, in P, five compounds with the highest OAV were identified, which were 2-decenal, 2-nonenal, nonanal, 2-undecenal, and octanal, and these are mainly oxidized from oleic and linoleic acid. In this sense, 2-Decenal, 2-Nonenal, and 2-Undecenal produce citrus fruit, sweet, vegetable, and herbaceous notes, among others. Octanal emits green, meaty, fresh, fruity, and grassy notes, whereas nonanal contributes fruity and sweet aroma notes as well as an unpleasant rancid pungent odor [40]. Thus, these compounds might contribute greatly to the overall aroma of chito by imparting green, fatty, fresh, and grassy notes [47].

**Figure 2:**
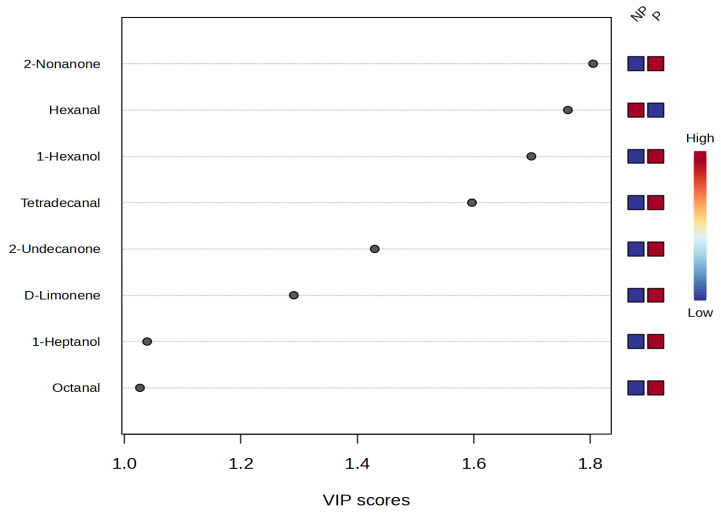
VIP scores of the volatile compounds in the chito (NP and P). NP: non-pressed (immediate consumption); P: pressed (for sale).

On the other hand, the variable importance in projection (VIP) of volatile compounds showed differences in their contributions across two different processes [non-pressed (NP) and pressed (P)] (Figure 2).

When the VIP of an odor component was equal to 1.0 or higher, it was used as a marker to distinguish the effect of pressing. Eight volatile compounds were considered as contributing significantly (VIP > 1), and aroma was contributed by 2-nonanone, hexanal, 1-hexanol, tetradecanal, 2-undecanone, D-limonene, 1-heptanol, and octanal. These compounds have shown odors related to fruity, floral, green, sweet, muttony, fatty, and rancid notes.

## 4. Conclusions

In this study, chito quality properties, including the volatile profile, were described by considering different sampling times. The results exhibited several differences between the two types of chito studied (non-pressed and pressed). This divergence in the results can be explained by the fact that the chito samples originated from different anatomical regions and manufacturing methods. Overall, the a_w_ values found in both types of chito are adequate for controlling meat deterioration with no significant pathogens detected. A low moisture content and a_w_ are important for the conservation of chito, which could be considered as an intermediate moisture meat product (a_w_ 0.60–0.90). Pressed chito showed higher hardness and higher oxidation values, which can influence the purchase decision. The addition of an antioxidant could inhibit oxidation reactions and improve the quality of chito. A total of 78 volatile compounds were detected by HS-SPME-GC-MS, among which aldehydes and alcohols were generally most abundant in pressed chito (P), indicating that lipid oxidation was the main pathway that influenced their generation. The presence of aldehydes with higher OAVs could have an impact on the overall aroma of chito. In addition, the analysis of volatile compounds showed that the analysed meat came from animals fed in an extensive system (rich in pastures), which imparted terpene-derived compounds to the meat (caryophyllene and D-limonene). Therefore, this study provides key insights into chito’s quality attributes and volatile profile, serving as a foundation for optimizing its processing and storage. Additionally, the findings may be valuable for the meat industry in enhancing the oxidative and sensory stability of the product.

## Figures and Tables

**Table 1:** Mean values of pH, a_w_, proximate composition (%), Heme iron (µg/g), NaCl (% of sodium chloride), and water soluble nitrogen (WSN; % of total nitrogen) of chito.

	Processing Year	Treatments		SEM	*P*-Level
		NP	P		
pH	2021	5.08 ^bB^	5.75 ^aA^	0.05	*
	2022	5.28 ^bA^	5.65 ^aB^	0.03	***
	2023	5.27 ^bA^	5.74 ^aA^	0.03	***
SEM		0.06	0.06		
*P*-level		**	**		
a_w_	2021	0.72 ^aA^	0.71 ^aA^	0.01	NS
	2022	0.68 ^aB^	0.68 ^aB^	0.03	NS
	2023	0.70 ^aAB^	0.69 ^aAB^	0.04	NS
SEM		0.04	0.05		
*P*-level		**	*		
Proximate composition					
Moisture	2021	33.16 ^aA^	30.17 ^aA^	1.87	NS
	2022	33.42 ^aA^	22.74 ^bB^	2.22	**
	2023	31.31 ^aA^	23.46 ^bB^	1.51	***
SEM		0.71	1.53		
*P*-level		NS	*		
Protein	2021	39.60 ^aA^	40.19 ^aA^	2.47	NS
	2022	38.27 ^aA^	41.98 ^aA^	1.74	NS
	2023	38.77 ^aA^	39.55 ^aA^	1.80	NS
SEM		1.41	1.45		
*P*-level		NS	NS		
Fat	2021	6.23 ^aB^	6.44 ^aB^	0.78	NS
	2022	11.59 ^bA^	15.03 ^aA^	1.90	**
	2023	4.82 ^bB^	9.34 ^aB^	2.14	*
SEM		1.55	1.56		
*P*-level		**	**		
Ash	2021	18.01 ^aA^	18.45 ^aA^	0.60	NS
	2022	15.86 ^aA^	17.37 ^aA^	0.44	NS
	2023	16.76 ^aA^	19.02 ^aA^	1.39	NS
SEM		0.44	0.94		
*P*-level		NS	NS		
Heme iron (µg/g)	2021	32.21 ^aC^	30.82 ^aB^	0.59	NS
	2022	81.82 ^aA^	49.30 ^bA^	2.14	***
	2023	57.56 ^aB^	20.82 ^bC^	2.23	***
		***	***		
		2.01	2.23		
NaCl	2021	10.39 ^bA^	10.93 ^aB^	0.10	*
	2022	10.55 ^bA^	11.21 ^aA^	0.28	**
	2023	10.74 ^bA^	11.07 ^aAB^	0.15	*
SEM		0.18	0.05		
*P*-level		NS	*		
WSN	2021	21.52 ^aA^	18.28 ^aA^	1.53	NS
	2022	10.31 ^aB^	8.37 ^aB^	2.04	NS
	2023	9.26 ^aB^	8.43 ^aB^	0.26	NS
SEM		2.07	1.79		
*P*-level		**	*		

NP: non-pressed (immediate consumption); P: pressed (for sale). SEM: standard error of means. ^a,b^: Means values within the same row with different letters are significantly different (*P* < 0.05). ^A–C^: Means values in the same column with different letters are significantly different (*P* < 0.05). *P*-level, Significance: NS: not significant; * (*P* ≤ 0.05); ** (*P* ≤ 0.01); *** (*P* ≤ 0.001).

**Table 2:** Mean values of instrumental color and metmyoglobin content of chito.

	Processing Year	Treatments		SEM	*P*-Level
		NP	P		
Color					
L*	2021	25.15 ^aA^	26.79 ^aA^	1.70	NS
	2022	25.92 ^aA^	20.11 ^aB^	0.73	NS
	2023	20.31 ^aB^	20.56 ^aB^	0.83	NS
SEM		0.99	1.10		
*P*-level		**	*		
a*	2021	7.48 ^aA^	6.16 ^bA^	0.75	*
	2022	5.92 ^aB^	5.76 ^aB^	0.73	NS
	2023	7.86 ^aA^	6.18 ^bA^	0.74	*
SEM		0.56	0.54		
*P*-level		**	*		
b*	2021	24.74 ^aA^	29.97 ^aA^	1.64	NS
	2022	29.41 ^aA^	30.09 ^aA^	0.49	NS
	2023	28.96 ^aA^	28.52 ^aA^	0.82	NS
SEM		0.78	0.98		
*P*-level		NS	NS		
Chroma	2021	30.91 ^aA^	26.23 ^aA^	1.71	NS
	2022	30.69 ^aA^	30.01 ^aA^	0.54	NS
	2023	30.81 ^aA^	29.26 ^aA^	0.76	NS
SEM		0.77	1.10		
*P*-level		NS	NS		
Hue	2021	72.00 ^bB^	77.04 ^aA^	1.28	*
	2022	78.90 ^aA^	79.14 ^aA^	0.56	NS
	2023	70.68 ^bB^	78.01 ^aA^	1.57	*
SEM		1.12	0.99		
*P*-level		**	NS		
Metmyoglobin (%)	2021	33.61 ^aB^	36.45 ^aB^	1.33	NS
	2022	36.32 ^bA^	53.21 ^aA^	1.24	***
	2023	30.66 ^aB^	32.02 ^aB^	0.80	NS
SEM		0.67	1.04		
*P*-level		***	***		

NP: non-pressed (immediate consumption); P: pressed (for sale). SEM: standard error of means. ^a,b^: Means values within the same row with different letters are significantly different (*P* < 0.05). ^A,B^: Means values in the same column with different letters are significantly different (*P* < 0.05). *P*-level, Significance: NS: not significant; * (*P* ≤ 0.05); ** (*P* ≤ 0.01); *** (*P* ≤ 0.001).

**Table 3:** Mean values of instrumental measurement texture of chito.

	Processing Year	Treatments		SEM	*P*-Level
		NP	P		
Texture profile analysis					
Hardness (N)	2021	55.39 ^bB^	223.67 ^aB^	55.19	*
	2022	154.4 ^bA^	574.01 ^aA^	84.31	***
	2023	75.75 ^bB^	328.01 ^aAB^	77.86	*
SEM		29.55	53.55		
*P*-level		**	***		
Springiness	2021	0.54 ^aA^	0.66 ^aA^	2.91	NS
	2022	0.59 ^aA^	0.69 ^aA^	2.10	NS
	2023	0.69 ^aA^	0.56 ^aA^	3.28	NS
SEM		3.21	3.09		
*P*-level		NS	NS		
Cohesiveness	2021	0.56 ^bA^	0.63 ^aA^	3.52	NS
	2022	0.59 ^aA^	0.69 ^aA^	4.27	NS
	2023	0.72 ^aA^	0.57 ^aA^	5.08	NS
SEM		3.35	3.38		
*P*-level		NS	NS		
Resilience	2021	0.19 ^aA^	0.20 ^aA^	1.24	NS
	2022	0.09 ^aB^	0.06 ^aB^	2.19	NS
	2023	0.09 ^aB^	0.05 ^aB^	2.64	NS
SEM		1.74	2.07		
*P*-level		*	**		

NP: non-pressed (immediate consumption); P: pressed (for sale). SEM: standard error of means. ^a,b^: Means values within the same row with different letters are significantly different (*P* < 0.05). ^A,B^: Means values in the same column with different letters are significantly different (*P* < 0.05). *P*-level, Significance: NS: not significant; * (*P* ≤ 0.05); ** (*P* ≤ 0.01); *** (*P* ≤ 0.001).

**Table 4:** Mean values of microbial counts (log CFU/g) of chito.

	Processing Year	Treatments		SEM	*P*-Level
		NP	P		
TMBC	2021	5.92 ^aB^	5.95 ^aB^	0.13	NS
	2022	6.68 ^aA^	7.00 ^aA^	0.20	NS
	2023	6.62 ^aA^	6.10 ^aB^	0.16	NS
SEM		0.11	0.19		
*P*-level		**	*		
LAB	2021	5.50 ^aA^	5.68 ^aA^	0.29	NS
	2022	4.56 ^aA^	4.91 ^aA^	0.63	NS
	2023	5.57 ^aA^	6.54 ^aA^	0.65	NS
SEM		0.50	0.39		
*P*-level		NS	NS		

CFU: colony-forming units. NP: non-pressed (immediate consumption); P: pressed (for sale). SEM: standard error of means. TMBC: Total Mesophilic Bacteria Count; LAB: Lactic Acid Bacteria. ^a^: Means values within the same row with different letters are significantly different (*P <* 0.05). ^A,B^: Means values in the same column with different letters are significantly different (*P <* 0.05). *P*-level, Significance: NS: not significant; * (*P* ≤ 0.05); ** (*P ≤* 0.01).

**Table 5:** The content of identified volatile compounds found in chito (NP and P) and their identification parameters.

					Concentration (µg/kg)					
Compound	RT (min)	Chemical Formula	Odor Description ^a^	CAS#	NP 2021	P 2021	NP 2022	P 2022	NP 2023	P 2023
Aldehydes										
Hexanal	3.72	C_6_H_12_O	Green leaves, fresh grass, fatty, rancid, unpleasant, tallowy, muttony	66-25-1	2.21	1.32	2645.61	975.37	829.6	434.85
Benzaldehyde	4.62	C_7_H_6_O	Bitter almond, almond, burnt sugar, roasted pepper, nutty	100-52-7	32.84	52.6	nd	nd	nd	nd
Heptanal	4.9	C_7_H_14_O	Potato, pleasant meaty notes, nutty, fruity green, aldehyde, fatty	111-71-7	nd	nd	788.22	839.55	520.57	533.24
Octanal	6.02	C_8_H_16_O	Green, citrus, lemon, meaty, fresh, fruity, grass, fatty	124-13-0	3.38	9.61	455.61	784.73	556.67	621.17
2-Octenal	6.7	C_8_H_14_O	Grilled meat, peanut cake, fat green leaf, floral	2548-87-0	nd	nd	128.69	146.43	141.01	112.37
Benzaldehyde, 4-methoxy-	6.98	C_8_H_8_O_2_	Similar hawkthorn	123-11-5	2.48	2.59	nd	nd	nd	nd
Nonanal	7.1	C_9_H18O	Fruity, sweet, pleasant meaty notes, citrus, green, citronella, grass, fat, waxy	124-19-6	26.54	20.86	1361.95	2067.41	1517.25	1808.72
2-Nonenal	7.71	C_9_H_16_O	Fruity–floral, vegetable, herbaceous, and/or chemical, earthy, fermented, burnt	18829-56-6	nd	nd	401.36	551.45	557.66	475.66
Decanal	8.13	C_10_H_20_O	Green, fishy, fatty, rancid, meaty, burnt, soap, orange peel, tallow	112-31-2	3.22	nd	165.74	217.16	153.83	123.99
2-Decenal	8.7	C_10_H_18_O	Fruity–floral, vegetable, herbaceous, citrus fruit, lemon, mint	3913-81-3	nd	nd	1262.19	1554.77	1031.31	1285.06
2-Undecenal	9.62	C_11_H_20_O	Sweet	2463-77-6	nd	nd	1763	1955.27	870.76	1574.87
Undecanal	9.09	C_11_H_22_O	Floral, green, mild	112-44-7	nd	nd	110.06	125.32	61.95	116.77
Benzaldehyde, 4-pentyl-	10.11	C_12_H_16_O	Sweet, woody	77961-30-1	3.26	2.56	nd	nd	nd	nd
Tridecanal	10.5	C_13_H_26_O	Fatty, sweet	629-62-9	nd	nd	216.23	236.15	95.49	188.64
Tetradecanal	11.66	C_14_H_28_O	Roasted, fried	629-59-4	nd	nd	279.07	214.57	116.05	579.44
Pentadecanal	12.21	C_15_H_30_O	Waxy	629-62-9	3.96	7.14	122.57	58.81	54.22	222.25
Hexadecanal	13.14	C_16_H_32_O	Fatty	629-80-1	3.76	51.09	4470.65	875.28	1058.8	11,980.47
9-Octadecenal	14.29	C_18_H_34_O	Fatty, green	5090-41-5	nd	nd	117.12	13.76	26.27	385.9
Octadecanal	14.58	C_18_H_36_O	Fatty, candle	638-66-4	nd	nd	65.08	137.77	156.43	4439.1
**Ketones**										
2-Heptanone	4.77	C_7_H_14_O	Fruity, citrus, grapefruit, limonene, floral, spicy cinnamon, spicy, blue cheese, acorn, soapy	110-43-0	nd	nd	256.95	136.35	56.64	90.71
2-Nonanone	6.98	C_9_H_18_O	Blue cheese, fruity, floral, flower petal	821-55-6	nd	nd	176.85	219.15	88.57	282.08
2-Decanone	8	C_10_H_20_O	Ethereal, butter, spicy, blue cheese, heavy, sweet	693-54-9	nd	nd	234.65	191.87	118.86	228.37
2-Undecanone	8.97	C_11_H_22_O	Fruity, fatty	112-12-9	nd	nd	246.98	389.7	129.85	228.83
Geranylacetone	10.39	C_13_H_22_O	Floral, rose, sweet	3796-70-1	nd	nd	145.64	111.04	58.82	117.94
2-Pentadecanone	12.29	C_15_H_30_O	Fatty, sweet	2345-28-0	nd	28.52	91.85	68.31	48.55	338.67
2-Heptadecanone	13.72	C_17_H_34_O	Waxy, floral	13922-62-8	nd	nd	101.41	71.61	56.71	466.24
**Alcohols**										
1-Hexanol	4.58	C_6_H_14_O	Green, sweet, spicy notes	111-27-3	nd	nd	43.19	95.17	53.16	70.52
Benzyl alcohol	5.17	C_7_H_8_O	Sweet, Grass	100-51-6	3.84	9.42	nd	nd	nd	nd
1-Heptanol	5.72	C_7_H_16_O	Woody, oily, floral	111-70-6	nd	2.57	158.51	225.89	170.86	231.16
1-Octen-3-ol	5.79	C_8_H_16_O	Mushroom-like, earthy, grass	3391-86-4	1.68	2	334	379.18	262.71	314.99
1-Octanol	6.79	C_8_H_18_O	Sharp, fatty, waxy, orange-rose, sweet	111-87-5	nd	nd	267.07	355.33	265.15	281.82
1-Dodecanol	7.39	C_12_H_26_O	Wax, sweet	112-53-8	nd	nd	54.22	88.2	136.49	54.77
2-Undecen-1-ol	8.82	C_11_H_22_O	Waxy, citrus-like	112-42-5	nd	nd	72.51	127.09	49.83	93.7
6-Pentadecen-1-ol	12.06	C_15_H_30_O	Waxy, green, oily	14652-30-5	nd	nd	53.14	41.22	108.63	148.39
Hexadecanol		C_16_H_34_O	Waxy, floral, fatty	36653-82-4	nd	nd	451	208.36	204.77	1166.33
**Acids**										
Acetic acid	2.52	C_2_H_4_O_2_	Pungent, acidic, cheesy, vinegar	64-19-7	7.59	nd	nd	nd	nd	nd
Hexanoic acid	5.01	C_6_H_12_O_2_	Goaty, pungency, rancid, cheese, fatty, sweaty, sour	142-62-1	5.25	8.37	nd	nd	nd	nd
Octanoic acid	7.56	C_8_H_16_O_2_	Rancid, fatty, coconut-like, vomit, cheese, rotten meat, waxy, sweaty	124-07-2	7.2	6.57	nd	nd	nd	nd
Benzoic acid	8.4	C_7_H_6_O_2_	Sharp, sweet, balsamic	65-85-0	6.64	nd	nd	nd	nd	nd
n-Decanoic acid	9.82	C_10_H_20_O_2_	Rancid, oily	334-48-5	2.89	14.67	nd	nd	nd	nd
Undecanoic acid	10.57	C_11_H_22_O_2_	Waxy, citrus-like	112-37-8	1.31	2.2	nd	nd	nd	nd
Lauric acid	11.32	C_12_H_24_O_2_	Soapy, fatty, sweet	143-07-7	7.06	3.24	nd	nd	nd	nd
Tridecanoic acid	12.07	C_13_H_26_O_2_	Waxy, oily	638-53-9	9.38	nd	nd	nd	nd	nd
Myristic acid	12.82	C_14_H_28_O_2_	Fatty, waxy	544-63-8	14.52	10.81	260.65	522.11	83.55	579.01
Pentadecanoic acid	13.53	C_15_H_30_O_2_	Oily, waxy	1002-84-2	4.41	nd	nd	nd	nd	nd
Palmitoleic acid	14.06	C_16_H_30_O_2_	Waxy, oily	373-49-9	nd	nd	104.91	163.07	19.78	277.53
Palmitic acid	14.24	C_16_H_32_O_2_	Fatty, waxy, mild	57-10-3	nd	nd	1527.09	1731.92	153.63	4556.27
Oleic acid	15.34	C_18_H_34_O_2_	Fatty, oily	112-80-1	nd	nd	362.36	452.32	234.89	1425.92
Stearic acid	15.45	C_18_H_36_O_2_	Waxy, fatty	57-11-4	nd	nd	135.68	257.99	34.22	838.1
**Terpenes**										
p-Cymene	6.29	C_10_H_14_	Sweet, citrus, weak, spicy herbaceous, fresh	99-87-6	nd	nd	43.05	51.22	21.58	24.81
D-Limonene	6.34	C_10_H_16_	Sweet, fruity, citrus, lemon	5989-27-5	nd	nd	45.68	125.33	43.11	30.99
Menthene	7.38	C_10_H_18_	Minty, herbal	5502-88-5	nd	nd	43.96	63.69	51.76	80.93
Cubenol	9.53	C_15_H_26_O	Woody, sweet	502-99-8	nd	nd	112.73	121.74	80	62.23
Copaene	9.81	C_15_H_24_	Woody, spicy	3856-25-5	nd	nd	118.12	134.73	116.61	126.93
*β*-Bourbonene	9.87	C_15_H_24_	Woody spicy, sickly sweet, wallflowers, balsamic	5208-59-3	nd	nd	167.79	65.83	133.79	171.96
Isocaryophillene	10.1	C_15_H_24_	Spicy, herbal	6753-98-6	nd	nd	83.64	85.11	73.95	40.78
*α*-Cedrene	10.19	C_15_H_24_	Woody	469-61-4	nd	nd	60.24	49.95	30.74	43.95
Caryophyllene	10.22	C_15_H_24_	Spicy, peppery	87-44-5	9.87	3.25	216.68	341.57	310.82	123.06
Caryophylladienol	10.28	C_15_H_26_O	Woody, sweet		nd	nd	38.74	42.13	40.16	21.62
*α*-Humulene	10.51	C_15_H_24_	Woody, hop-like, musty earthy	6753-98-6	nd	nd	122.23	106.25	41.03	97.15
Eudesmene	10.79	C_15_H_24_	Woody, balsamic	5153-27-3	nd	nd	48.41	44.4	26	16.59
Isopatchoulane	10.84	C_15_H_26_	Earthy, woody	10094-06-9	nd	nd	157.86	154.38	125.78	167.37
*γ*-Muurolene	10.99	C_15_H_24_	Woody, earthy, herbal	473-15-4	nd	nd	27.45	nd	16.33	nd
*α*-Calacorene	11.23	C_15_H_24_	Woody, spicy, herbal	1131-62-0	nd	nd	89.45	52.85	126.86	136.11
Caryophyllene oxide	11.61	C_15_H_24_O	Woody, spicy, green	1139-30-6	nd	nd	32.34	36.43	18.8	30.78
Squalene	13.48	C_30_H_50_	Odorless to slightly oily	111-02-4	nd	nd	55.24	284.96	22.29	171.54
**Hydrocarbons**										
Heptane	2.85	C_7_H_16_	Hydrocarbon (weak), sweet, gasoline-like, light petroleum	142-82-5	nd	nd	25.78	100.45	23.43	35.06
Octane	3.78	C_8_H_18_	Hydrocarbon (weak), fatty, solvent	111-65-9	nd	nd	324.67	194.8	212.97	84.55
Nonane	4.86	C_9_H_20_	Sweet, mild gasoline odor	111-84-2	nd	nd	13.74	34.64	9.66	7.98
Pentadecane	10.72	C_15_H_32_	Waxy, oily, mild	629-62-9	nd	nd	245.72	221.3	98.93	236.65
Hexadecane	11.5	C_16_H_34_	Odorless or very faint hydrocarbon smell	544-76-3	nd	nd	161.51	187.58	35.16	187.33
Heptadecane	12.26	C_17_H_36_	Odorless	629-78-7	nd	nd	138.78	96.3	33.09	187.5
Octadecane	12.98	C_18_H_38_	Odorless	593-45-3	nd	nd	90.29	102.84	32.98	205.2
Nonadecane	13.66	C_19_H_40_	Odorless	629-92-5	nd	nd	280.88	62.41	51.36	910.16
Phytane	13.06	C_20_H_42_	Odorless	638-36-8	nd	nd	127.54	93.26	48.42	366.28
**Phenols**										
Phenol	11.31	C_6_H_6O_	Phenolic medicinal	108-95-2	1.69	2.93	nd	nd	nd	nd
*p*-Cresol	13.06	C_7_H_8O_	Phenolic, horse-stable-like	106-44-5	7.51	4.26	nd	nd	nd	nd
*m*-Cresol	13.07	C_7_H_8O_	Tar, medicinal, phenolic	108-39-4	23.38	6.13	nd	nd	nd	nd

RT: retention time. NP: non-pressed (immediate consumption); P: pressed (for sale). CAS#: CAS number. nd: The compounds not detected in the sample. ^a^ Odor description from literature [18,37,38,39,40,41,42,43].

**Table 6:** Odor activity value (OAV) of the aroma compounds detected in chito (NP and P).

			OAV					
Volatile Compound	Odor Threshold (µg/kg)	Ref.	NP 2021	P 2021	NP 2022	P 2022	NP 2023	P 2023
**Aldehydes**								
Hexanal	5	[38,39]	0.44	0.26	529.12	195.07	165.92	86.97
Heptanal	2.8	[38,39]	nd	nd	281.51	299.84	185.92	190.44
Octanal	0.59	[38,52]	5.73	16.29	772.22	1330.0	943.51	1052.8
2-Octenal	3	[53]	nd	nd	42.90	48.81	47.00	37.46
Nonanal	1.1	[38,53]	24.13	18.96	1238.14	1879.46	1379.32	1644.29
2-Nonenal	0.19	[38,53]	nd	nd	2112.42	2902.37	2935.05	2503.47
Decanal	3	[38,39]	1.07	nd	55.25	72.39	51.28	41.33
2-Decenal	1	[38]	nd	nd	4207.30	5182.57	3437.70	4283.53
2-Undecenal	0.78	[38]	nd	nd	1259.29	1396.62	621.97	1124.91
Tridecanal	10	[38]	nd	nd	21.62	23.62	9.55	18.86
Tetradecanal	110	[38]	nd	nd	2.54	1.95	1.06	5.27
**Ketones**								
2-Heptanone	140	[38,52]	nd	nd	1.84	0.97	0.40	0.65
2-Nonanone	41~82	[38]	nd	nd	4.31	5.35	2.16	6.88
2-Decanone	8~41	[38]	nd	nd	28.27	23.12	14.32	27.51
2-Undecanone	5.5	[38]	nd	nd	44.91	70.85	23.61	41.61
Geranylacetone	0.06	[43]	nd	nd	2.43	1.85	0.98	1.97
**Alcohols**								
1-Hexanol	5.6	[39]	nd	nd	7.71	16.99	9.49	12.59
1-Heptanol	5.4	[39]	nd	0.48	29.35	41.83	31.64	42.81
1-Octen-3-ol	1.5	[38,39]	1.12	1.33	222.67	252.79	175.14	209.99
1-Octanol	120	[38]	nd	nd	2.12	2.82	2.11	2.24
1-Dodecanol	16	[38]	nd	nd	3.39	5.51	8.53	3.42
**Terpenes**								
*p*-Cymene	5.01	[38]	nd	nd	8.59	10.22	4.31	4.95
D-Limonene	10	[53]	0.00	0.00	1.34	3.69	1.27	0.91
Caryophyllene	0.064	[43]	0.15	0.05	3.39	5.34	4.86	1.92

Ref.: References. NP: non-pressed (immediate consumption); P: pressed (for sale). nd: the compounds not detected in the sample.

## Data Availability

The original contributions presented in the study are included in the article/Supplementary Materials. Further inquiries can be directed to the corresponding author.

## Supplementary Materials

The following supporting information can be downloaded at: https://www.mdpi.com/article/10.3390/foods14132341/s1, Figure S1: Heatmap of volatile compounds identified in the chito (NP and P) using headspace solid phase microextraction combined with gas chromatography-mass spectrometry (HS-SPME GC-MS) analysis. The blocks of colours from red to blue indicated that the compounds were present from higher to lower levels. NP: non-pressed (immediate consumption); P: pressed (for sale).
