# Supplementary material for: Physicochemical Properties and Volatile Profile of Chito: A Traditional Dry-Cured Goat Meat Product
Source: Foods. 2025 Jul 1;14(13):2341. doi: 10.3390/foods14132341 (PMC12249266; doi:10.3390/foods14132341)
Supplement: Supplementary file 1 [file foods-14-02341-s001.zip › Figure S1 Heatmap of volatile compounds identified in the chito.pdf]

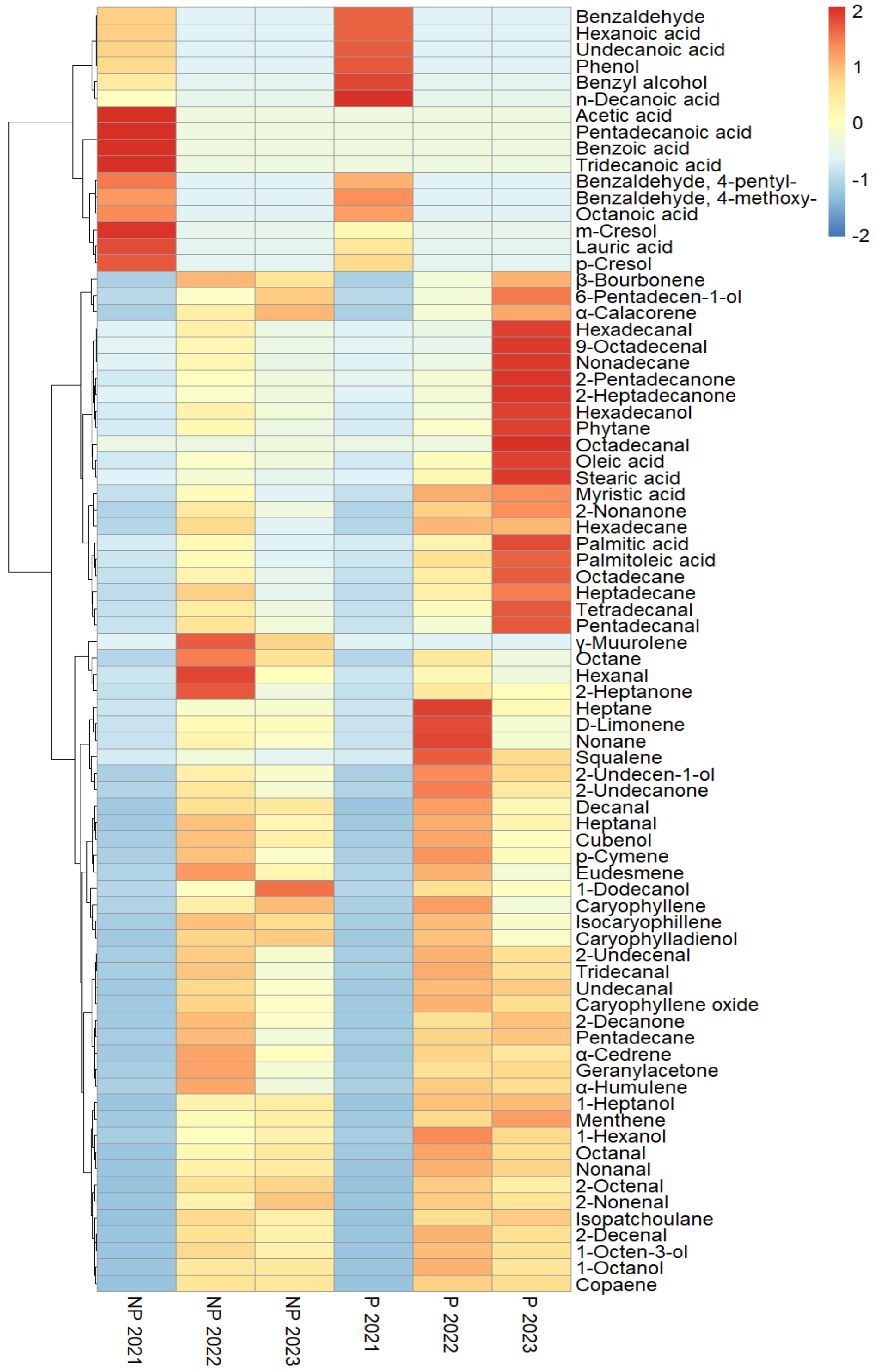

Figure. S1. Heatmap of volatile compounds identified in the chito (NP and P) using headspace solid phase microextraction combined with gas chromatography-mass spectrometry (HS-SPME GC-MS) analysis. The blocks of colours from red to blue indicated that the compounds were present from higher to lower levels. NP: non-pressed (immediate consumption); P: pressed (for sale).
